# Global cortical activity predicts shape of hand during grasping

**DOI:** 10.3389/fnins.2015.00121

**Published:** 2015-04-09

**Authors:** Harshavardhan A. Agashe, Andrew Y. Paek, Yuhang Zhang, José L. Contreras-Vidal

**Affiliations:** ^1^Noninvasive Brain-Machine Interface Systems Lab, Electrical and Computer Engineering, University of HoustonHouston, TX, USA; ^2^Hyperspectral Image Analysis Lab, Department of Electrical and Computer Engineering, University of HoustonHouston, TX, USA

**Keywords:** grasping, electroencephalography, decoding, brain-machine interfaces

## Abstract

Recent studies show that the amplitude of cortical field potentials is modulated in the time domain by grasping kinematics. However, it is unknown if these low frequency modulations persist and contain enough information to decode grasp kinematics in macro-scale activity measured at the scalp via electroencephalography (EEG). Further, it is unclear as to whether joint angle velocities or movement synergies are the optimal kinematics spaces to decode. In this offline decoding study, we infer from human EEG, hand joint angular velocities as well as synergistic trajectories as subjects perform natural reach-to-grasp movements. Decoding accuracy, measured as the correlation coefficient (r) between the predicted and actual movement kinematics, was *r* = 0.49 ± 0.02 across 15 hand joints. Across the first three kinematic synergies, decoding accuracies were *r* = 0.59 ± 0.04, 0.47 ± 0.06, and 0.32 ± 0.05. The spatial-temporal pattern of EEG channel recruitment showed early involvement of contralateral frontal-central scalp areas followed by later activation of central electrodes over primary sensorimotor cortical areas. Information content in EEG about the grasp type peaked at 250 ms after movement onset. The high decoding accuracies in this study are significant not only as evidence for time-domain modulation in macro-scale brain activity, but for the field of brain-machine interfaces as well. Our decoding strategy, which harnesses the neural “symphony” as opposed to local members of the neural ensemble (as in intracranial approaches), may provide a means of extracting information about motor intent for grasping without the need for penetrating electrodes and suggests that it may be soon possible to develop non-invasive neural interfaces for the control of prosthetic limbs.

## Introduction

Grasping is one of the most fundamental ways humans interact with the world, allowing us to manipulate and interact with objects around us. The kinematics of grasping and the neuroscience underlying the smooth and continuous control of the hand and fingers have been studied extensively (Jeannerod, 1984; Santello et al., 2002; Castiello, 2005), and experiments have shown modulation in neural spiking activity associated with various stages of grasping (Rizzolatti et al., 1988; Murata et al., 1997; Bansal et al., 2011). PET and fMRI experiments show the involvement of widely distributed brain areas during a self-initiated grasping movement (Castiello, 2005). Proximal and distal upper extremity movement information has been shown to be encoded as the power in various frequency bands in cortical field potentials at various spatial scales, such as local field potentials (LFPs), electrocorticography (ECoG), electroencephalography (EEG), and magnetoencephalography (MEG) (Ball et al., 2008; Kubánek et al., 2009; Waldert et al., 2009; Zhuang et al., 2010; Pistohl et al., 2012). More recently, researchers have shown that information is also encoded in the time-domain amplitudes of these fields in the lowest frequency band (0–5 Hz) (Bradberry et al., 2009, 2010; Kubánek et al., 2009; Acharya et al., 2010; Bansal et al., 2011; Mollazadeh et al., 2011; Hall et al., 2014). A summary of results from grasp decoding studies is shown in Table 1. It remains unclear if these amplitude modulations contain enough information to be able to infer the dexterous movement of the fingers during grasping, at the macro scale of scalp EEG.

**Table 1:** **Summary of grasp decoding studies**.

**Behavioral task**	**Decoded kinematics**	**Decoding accuracy**	**Signal modality; features; subjects**	**References**
3D Reach-to-grasp	Finger joint angles	Monkey C: *r* = 0.72 Monkey G: *r* = 0.74	Microelectrodes; neuron firing rates; Monkeys	Vargas-Irwin et al., 2010
3D Reach-to-grasp	Grasp aperture	Delta: *r* = 0.46 Gamma: *r* = 0.62	Microelectrodes; LFP frequency bands; Monkeys	Zhuang et al., 2010
3D Reach-to-grasp	Grasp aperture	Position: *r* = 0.65 velocity: *r* = 0.75	Microelectrodes; LFP 0.3–2 Hz; Monkeys	Bansal et al., 2011
Slow grasping motion	Finger joint angle PC	*r* = 0.52	ECoG; 2 s moving average filter; Human patients	Acharya et al., 2010
Repetitive individual finger flexion and extension	MCP joint angles	Thumb: *r* = 0.56 Index: *r* = 0.60 Middle: *r* = 0.54 Ring: *r* = 0.50 Little: *r* = 0.42	ECoG; 100 ms average window; frequency bins from 8 to 175 Hz; Human patients	Kubánek et al., 2009
Repetitive finger taps	Index finger MCP joint angle	*r* = 0.36	EEG; 0.1–3 Hz; Human subjects	Paek et al., 2014
3D Reach-to-grasp	MCP joint angles	*r* = 0.76	EEG; 0.1–1 Hz with genetic algorithm; Human subjects	Agashe and Contreras-Vidal, 2011
3D Reach-to-grasp	Precision vs. Power grasp classification	Classification accuracy = 88%	ECoG; 0–5 Hz, Human subjects	Pistohl et al., 2012
3D reach-to-grasp	Finger joint velocities and their PCs	PC1 *r* = 0.59 PC2 *r* = 0.47 PC3 *r* = 0.32	EEG; 0.1–1 Hz; Human subjects	Current study

LFP modulations have been shown to contain information about grasping movements not just in the primary motor cortex, but in a multitude of other brain areas as well (Bansal et al., 2011; Mollazadeh et al., 2011; Hall et al., 2014). While it is clear that a widely distributed network involving pre-frontal, sensori-motor as well as visuo-motor areas in both hemispheres is responsible for the control of self-initiated grasping actions (Matsumura et al., 1996; Rizzolatti et al., 1996), the characterization of scalp-level neural representations of these areas remains unknown. Recent findings revealed cyclic activity in motor cortex LFP signals locked to “submovements” (Hall et al., 2014). Understanding how these delta band (0–4 Hz) submovements combine to yield functional motion may provide clues to the origins of delta-band activity and why they encode upper limb movement information (Hall et al., 2014).

Apart from the neuroscience community, being able to decode grasping kinematics is of great interest to the brain-machine interface (BMI) community. Upper limb amputation, stroke, or severe spinal cord injury result in loss or significant reduction in bimanual motor function and dexterous hand movements in the affected limb(s). Improved upper extremity function is the leading requirement among tetraplegics (Snoek et al., 2004) and other clinical populations with impaired hand function. Control of hand prosthetics with peripheral signals such as intramuscular/surface electromyography (sEMG) (Cipriani et al., 2011, 2014) and targeted muscle re-innervation (Kuiken et al., 2009) show promise. BMI which extract movement intent from brain activity and control external devices are another possible strategy to regain hand function (Birbaumer, 2006; Lebedev and Nicolelis, 2006) while also tracking plasticity in the brain. Current upper limb neuroprosthetics restore some degree of functional ability, but fail to approach the ease of use and dexterity of the natural hand, particularly for grasping movements. Control of an anthropomorphic robotic arm with intracortically recorded neural activity was recently shown to be possible (Collinger et al., 2012; Hochberg et al., 2012; Wodlinger et al., 2015). These invasive BMI systems are able to extract intended arm position and movement in space, along with a control over opening and closing a grasp. However, the multitude of grasp types required in activities of daily living require a detailed level of control over manual dexterity and grasp posture. Further, the inherent risks associated with surgery required to implant electrodes, along with the long-term stability of recorded signals, is of concern (Schultz and Kuiken, 2011). Current approaches to non-invasive BMIs typically require the user to learn to control the power in their sensorimotor mu-rhythms (specific frequency bands usually centered around 10 and 22 Hz) (Wolpaw and McFarland, 2004; McFarland et al., 2010). Here we show that it is feasible to extract detailed information on intended grasping movements to various objects in a natural, intuitive manner, from a plurality of scalp EEG signals.

Research shows that to manipulate the large number of joints available in the hand during grasping, the motor system controls an inherently low-dimensional manifold called the synergy space (Santello et al., 1998, 2002; Vinjamuri et al., 2010). A common approach to identifying these movement synergies is by decomposing joint angle velocities into their Principal Components (PCs) (Santello et al., 1998). The kinematic space (joint angle velocities vs. movement synergies) that is optimally encoded in cortical field potentials remains unknown. In this study, five human subjects performed self-initiated and natural reach-to-grasp movements to five common objects while EEG and hand kinematics were recorded simultaneously. We selected five objects requiring distinct types of grasps: a soda can (cylindrical power grasp), a CD (whole hand circular grasp), a credit card (lateral precision grasp), a small coin (precision pinch grasp), and a screwdriver (tool power grasp) (Santello et al., 1998). In an offline analysis, we reconstructed the trajectories of the hand in both joint angle velocity and synergy (PC) spaces during the grasping movement. The decoding was performed with a linear regression model with lags (i.e., time delay between EEG and decoded kinematics; see Materials and Methods). Notably, the time-domain feature space, coupled with the linear decoder, requires that the EEG signals share the same frequency bandwidth as the movement kinematics. The majority of power in grasping movements performed by subjects in this study was concentrated in the 0.1–1 Hz band (see Materials and Methods), requiring that EEG be low-pass filtered at 1 Hz as well (see Materials and Methods).

## Materials and methods

### Data acquisition and experiment design

The Institutional Review Board (IRB) at the University of Houston approved this research. Five healthy, able-bodied right-handed volunteers (4 males, 1 female; ages 20–28 years) participated in this study after giving written informed consent. Whole head 64-channel EEG with a 10–20 system layout (Brain Vision LLC, USA) and hand kinematics were recorded simultaneously at 1000 Hz using BCI2000 software (Schalk et al., 2004), while participants performed an object grasping task. The trajectories of 18 hand joint angles were recorded with a data glove (CyberGlove Systems LLC, USA). The following 15 joint angles were recorded and used for further processing: metacarpo-phalangeal (MCP) and proximal inter-phalangeal (PIP) joints for the four fingers; carpo-metacarpal (CMC), metacarpo-phalangeal (MCP), and interphalangeal (IP) joint for the thumb; and abduction/adduction (ABD) between the fingers. In addition, three more joints were recorded but not used for further processing: flexion/extension and abduction/adduction of the wrist; and palm arch which measures the curvature across the palm. These joint angles were not used for further processing since the wrist orientation does not determine the finger posture relevant to grasping the object, and the palm arch sensor was not used due to low resolution over its limited range of motion (0–5°). An accelerometer mounted on the wrist was used to record hand transport in three subjects (subjects S1, S2, and S3).

During the behavioral task, subjects were seated behind a table with their hand resting on a push-button, which was used to detect movement onset and offset. In front of the push-button, objects were presented on a visually marked area on the table's surface. The distance between the object and the push-button were determined such that subjects were able to reach the objects comfortably. One of five objects (soda can, credit card, CD, US penny, and a screwdriver) was placed on the marked area in a pseudorandom sequence. Subjects were instructed to start each trial with a relaxed gesture on the switch, then self-initiate a reach and grasp movement to the object, followed by release and back to the resting position, at their preferred speed. The mean movement time was 1.9 ± 0.3 s across all subjects. Each subject performed 250 trials (50 trials per object), except subject S4 who performed 270 trials.

### EEG data preprocessing

**Figure 1:**
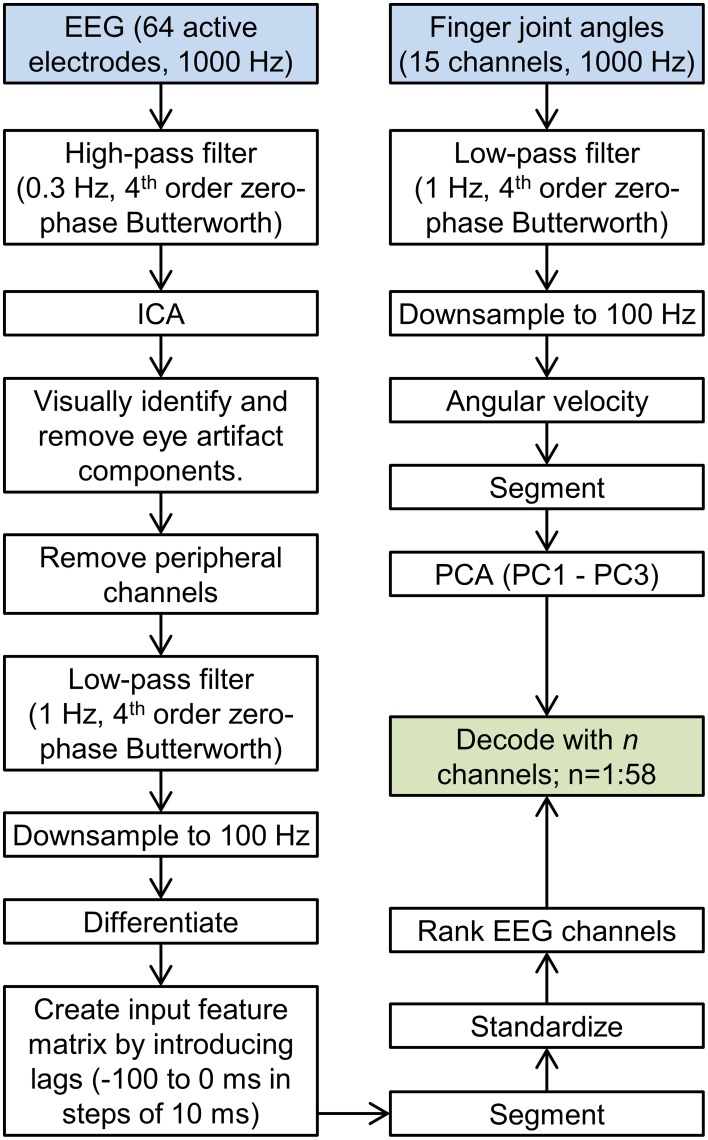
**Data processing flowchart**. The flowchart showing all data processing steps leading up to the decoding is shown. The left stream corresponds to EEG processing, and the right stream corresponds to kinematics processing.

All following analyses were performed in MATLAB (The MathWorks Inc., USA). Raw EEG data were detrended and high-pass filtered at 0.3 Hz with a zero-phase 4th order Butterworth filter to remove amplifier drift and ultra-low frequency components (Figure 1). Independent component analysis (ICA) was used to decompose the EEG into statistically independent components using the EEGLAB toolbox (Delorme and Makeig, 2004) after manually removing data segments corrupted by movement artifacts. Independent components corresponding to eye blinks and eye movements were identified and removed, followed by a projection back to the scalp EEG space. Six peripheral channels (M1, M2, TP9, TP10, PO9, and PO10) were excluded from further analysis. EEG data were low-pass filtered at 1 Hz with a zero-phase 4th order Butterworth filter. These filter cutoffs (0.3–1 Hz) were chosen based on previous findings (Bradberry et al., 2010; Agashe and Contreras-Vidal, 2011; Garipelli et al., 2013). EEG data were downsampled to 100 Hz and differentiated. Temporally lagged versions of EEG were concatenated along the third dimension, for a total of 11 lags (10 to 0 samples, corresponding to 100 to 0 ms in steps of 10 ms), resulting in an *n* × 58 × 11 data matrix, with the first dimension corresponding to time, the second dimension to EEG channels and the third dimension to lags. Continuous EEG was segmented into trials, from 400 ms before movement onset to 100 ms after movement offset and standardized by subtracting the mean and dividing by standard deviation across all trials.

### Kinematics data preprocessing

Fifteen hand joint angles were recorded at 1000 Hz synchronously with EEG data. The joint angle data were lowpass filtered at 1 Hz with a 4th order zero-phase Butterworth filter, followed by downsampling to 100 Hz (Figure 1). The change introduced due to filtering the kinematics was quantified using the signal-to-error ratio (*SER*) defined as $SER\left(y,y^{*}\right)=10\log_{10}\frac{Var\left(y\right)}{MSE\left(y,y^{*}\right)}$, where *y* is the raw kinematics, y^*^ is the filtered kinematics, *Var* denotes the variance and *MSE* is the mean square error. The mean *SER* across all blocks was found to be 15.5 ± 2.9 dB ensuring filter cutoffs were appropriate. Joint angles were then differentiated to yield angular velocities. Kinematics were segmented consistent with EEG (400 ms before movement onset to 100 ms after movement offset). Principal Component Analysis (PCA) was used to decompose the joint angular velocities into kinematic synergies, across all trials. The input to the PCA matrix consisted of an *n* × 15 matrix, where *n* is the sum of trial lengths. The first three synergies accounted for 90 ± 1% of the variance and were retained for decoding. Individually, the first three synergies accounted for 50 ± 1%, 29 ± 1%, and 10 ± 1% of the variance.

### Decoding

A Wiener filter was used to continuously decode joint angle velocity PCs:

$$PC_{i}\left[t\right]=\beta_{0i}+\sum_{n=1}^{N}\sum_{k=0}^{L}\beta_{nki}EEG_{n}\left[t-k\right]$$

where *PC_i_*[*t*] is the *i*th PC (*i* = 1, 2, 3 for the first three PCs), β_*nki*_ are the model parameters, *N* = 1 − 58 are the number of EEG channels used for decoding, *L* is the maximum time lag (100 ms) and *EEG_n_*[*t* − *k*] is the preprocessed EEG value of the *n*^th^ channel at time *t* − *k*.

Within each subject, an 8-fold cross validation procedure was employed to assess the decoding accuracy: data were divided into eight parts, with the *i*th part designated as testing data in the *i*th cross validation fold (a total of 8-folds). The remaining seven parts in a cross validation fold constituted the training data for that fold. For each cross-validation fold, model parameters were calculated on training data by minimizing the square error between the observed and model-estimated values for each PC. These model parameters were then applied to pre-processed EEG from the testing set to obtain a prediction of the PC value. We report the median correlation coefficient between the predicted and the observed PC values across all folds as the metric to assess decoding accuracy.

We evaluated the dependency of the decoding accuracy on the number of EEG channels used for the decoding process by the following procedure. Preprocessed EEG channels were ranked according to how well they were correlated with the three kinematic principal components (PCs), averaged over all lags. Specifically, the metric used for ranking was,

$$R_{n}=\frac{1}{L+1}\sum_{k=0}^{L}\sqrt{\beta_{nkPC1}^{2}+\beta_{nkPC2}^{2}+\beta_{nkPC3}^{2}},$$

where *R_n_* is the metric for the *nth* EEG channel and β are the regression parameters calculated over the entire dataset for each subject (Bradberry et al., 2010). The 8-fold cross validation decoding procedure was then performed iteratively using the best *N* EEG channels (*N* = 1 to 58) according to the channel ranking metric described above.

We assessed the contribution %*T_i_* at each lag *i* (*i* = 0 to −100 ms) as,

$$\%T_{i}=100\%\times \frac{\sum_{n=1}^{N}\sqrt{\beta_{nkPC1}^{2}+\beta_{nkPC2}^{2}+\beta_{nkPC3}^{2}}}{\sum_{k=0}^{L}\sum_{n=1}^{N}\sqrt{\beta_{nkPC1}^{2}+\beta_{nkPC2}^{2}+\beta_{nkPC3}^{2}}}.$$

We plotted the contributions from EEG channels at each lag on a scalp map to graphically assess the evolution of sensor contributions with time.

### Discrete classification

We quantified the information content in EEG by measuring its ability to discriminate between the grasp types with a multiple kernel learning (MKL) classifier (Rakotomamonjy et al., 2008; Gönen and Alpaydın, 2011), which is a multiple-kernel generalization of support vector machines (SVM). The key idea of MKL is to replace the single kernel in a SVM by a weighted linear combination of different basis kernels. The scalp was divided into 8 regions of interest (ROIs): left frontal (LF), right frontal (RF), left temporal (LT), right temporal (RT), left sensori-motor (LSM), right sensori-motor (RSM), left parietal-occipital (LPO), and right parietal-occipital (RPO). The combined kernel function *K* (***x_i_***, ***x_j_***) for input feature samples ***x_i_*** and ***x_j_*** was represented as

$$K\left(x_{i},\;x_{j}\right)=\sum_{m=1}^{M}d_{m}K_{m}(x_{i},\;x_{j}),$$

such that *d_m_* ≥ 0, and $\sum_{m=1}^{M}d_{m=1}$, where *M* = 24 is the number of basis kernels and *d_m_* is the weight for the *m^th^* basis kernel *K_m_*. Parameters *d_m_* were optimized through gradient descent on an SVM-based objective function according to “SimpleMKL” algorithm (Rakotomamonjy et al., 2008). Radial basis functions with relative width parameter σ = {5, 10, 15} were used as basis kernels for each of the 8 ROIs, resulting in a total of 24 basis kernels. This range of values was found to be reasonable after applying kernel alignment to an initial training set for each subject (Shawe-Taylor and Kandola, 2002). The input features for the discrete classifier were identical to the Wiener filter decoder detailed above, viz. low frequency time-domain EEG, lagged from 0 to 100 ms.

The information content in EEG was calculated as the reduction in entropy of the probability distribution over grasp types, due to the classifier output, given the EEG pattern (Quian Quiroga and Panzeri, 2009):

$$I=\sum_{PG,MG}P\left(PG,MG\right){\text{log}}_{2}\frac{P\left(PG,MG\right)}{P\left(PG\right)P\left(MG\right)},$$

where *I* is the information content in EEG about the grasp type; *P*(*PG*, *MG*) is the joint probability over predicted grasp type *PG* and measured grasp type MG, calculated from the confusion matrix of the classifier; *P*(*PG*) and *P*(*MG*) are the marginal probabilities. We calculated the information content from −1 to 3 s with respect to movement onset, in steps of 250 ms. For each time step, an 8-fold cross validation scheme was used.

## Results

### Both joint and synergy spaces can be decoded from scalp EEG

**Figure 2:**
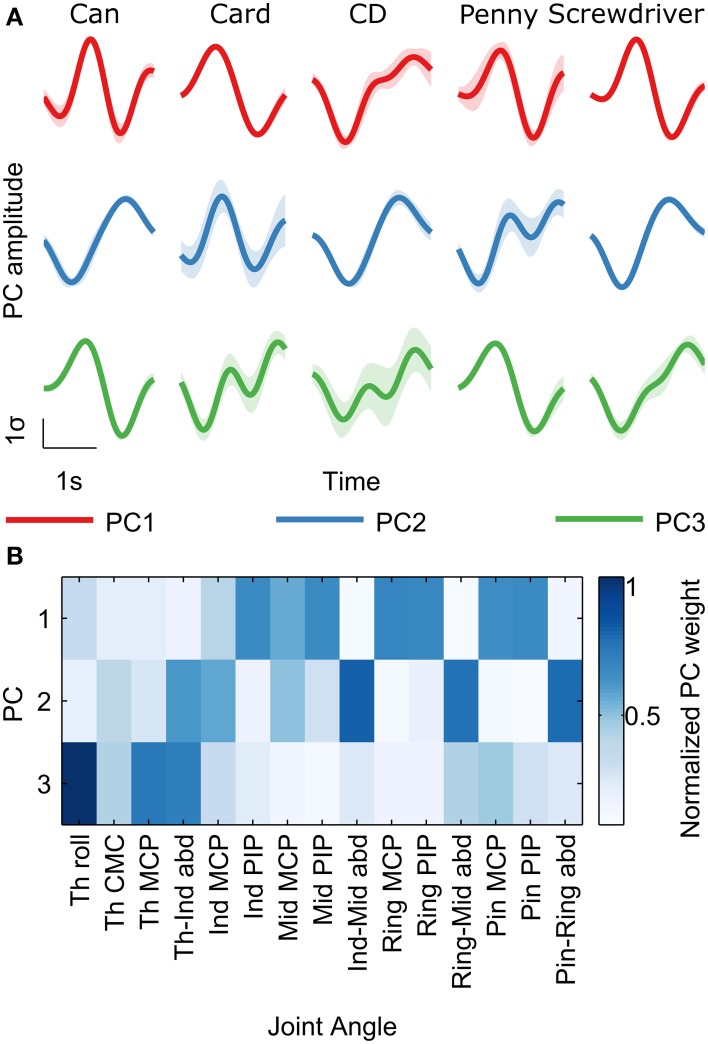
**Kinematic trajectories show synergies while grasping objects**. Principal Component Analysis (PCA) was performed on the 15 recorded joint angular velocities across all trials for each subject. **(A)** Mean principal component (PC) amplitudes (± s.e.m; shaded regions) are shown for the first three PCs for all objects in subject S5, from 400 ms before movement onset to 100 ms after movement offset. For display purposes, the different trial lengths for an object were rescaled in time to the mean trial length for that object. PC amplitudes from each trial were standardized to zero mean and unity standard deviation. **(B)** PC loading (weights) on the 15 joint angles are shown for the first three PCs. Darker colors represent larger weight magnitudes averaged across all subjects, normalized between zero and one. PC1 is highly loaded on the finger PIP and MCP joints representing grasp opening/closing motion. PC2 is loaded mainly on the abduction joints, representing the spreading motion of the hand. PC3 is loaded on the thumb joints, mainly rotation, and represents the independent movement of the thumb.

Previous studies point to both joint angular velocities as well as synergies as possible spaces in which the brain encodes grasping movement information (Kubánek et al., 2009; Acharya et al., 2010; Vargas-Irwin et al., 2010; Agashe and Contreras-Vidal, 2011; Pistohl et al., 2012). Here, we found high decoding accuracies for both joint angle velocities and their synergies. Movement synergies were calculated as the principal components (PCs) of joint angular velocities across all grasp types (Santello et al., 1998; Vinjamuri et al., 2010). We retained the first three PCs, which retained 90% of the variance. Figure 2 shows examples of PC trajectories across all objects and the visualization of PC loadings on each joint angle. PC1 was highly loaded on the finger PIP and MCP joints representing grasp opening/closing motion. PC2 was loaded mainly on the abduction joints, representing the spreading motion of the hand. PC3 was loaded on the thumb joints, mainly rotation, and represents the independent movement of the thumb.

**Figure 3:**
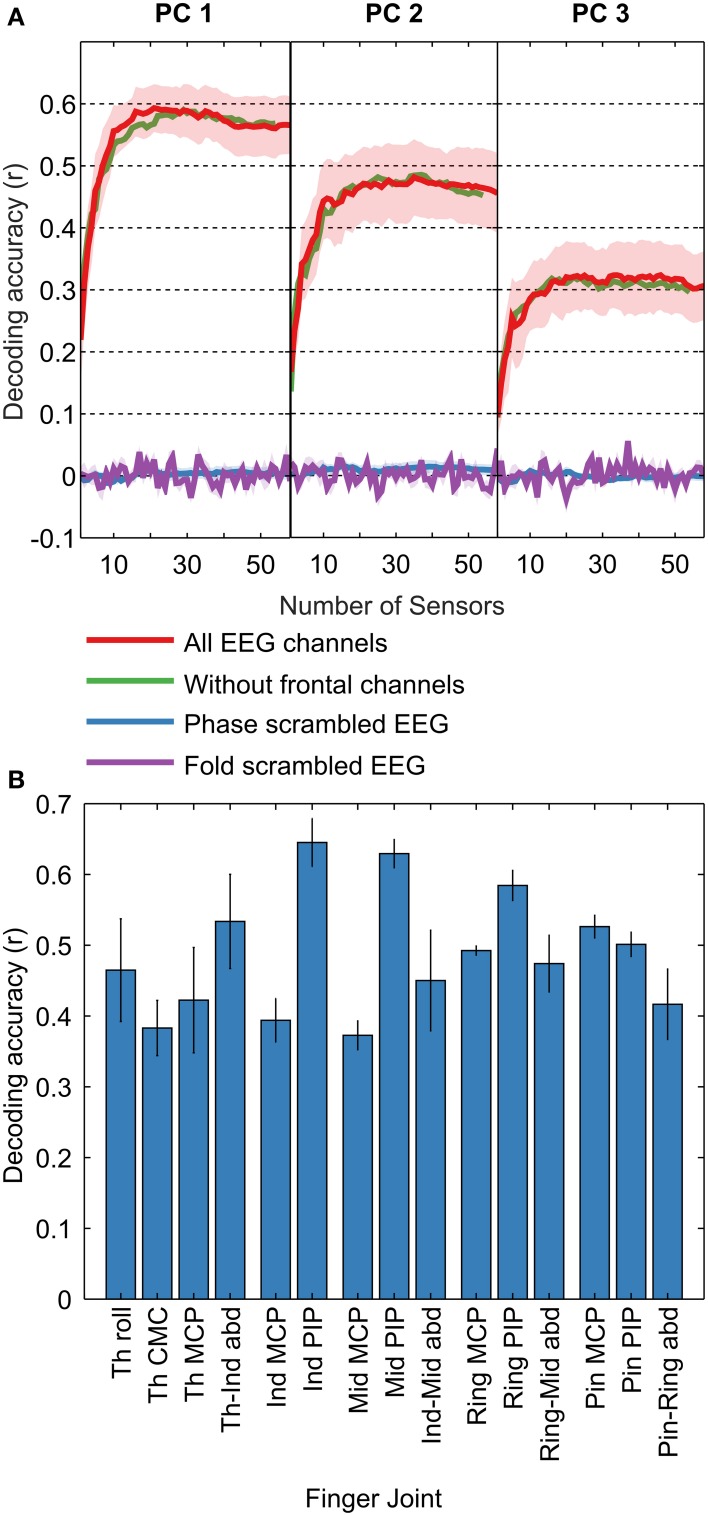
**Decoding Accuracies**. Decoding accuracies were calculated as the median across 8-folds for each of the 58 EEG sensor (channel) sets, for all subjects, for the first three PCs and the 15 joint angles. **(A)** Mean decoding accuracies across subjects are shown in red (± s.e.m; shaded regions) for PC1, PC2, and PC3. As the number of channels used in the decoding increases from 1 to 58, the curves shows a rapid increase followed by a slow decrease. To assess the validity of our results, we also calculated chance levels using two methods: (1) by scrambling the phase of the EEG and (2) by scrambling the EEG trial indices. The mean decoding accuracy (± s.e.m; shaded regions) for the “phase scrambled” and “fold scrambled” is shown in blue and purple traces, respectively. Decoding accuracies are seen to be far above chance levels, indicating the validity of our method. To assess the impact of ocular artifact on the decoding accuracy, we omitted 4 frontal EEG channels most affected by such artifacts and recalculated the decoding accuracy, shown by the green trace. The change in decoding accuracy on omitting the frontal channels is minimal, demonstrating the independence of our results on eye artifacts. **(B)** Decoding accuracy curves similar to panel **(A)** were calculated across EEG channel sets for each joint angle. The highest decoding accuracy for each curve is shown for each joint angle. In general, decoding accuracies were higher for finger PIP joints and lowest for MCP joints.

**Figure 4:**
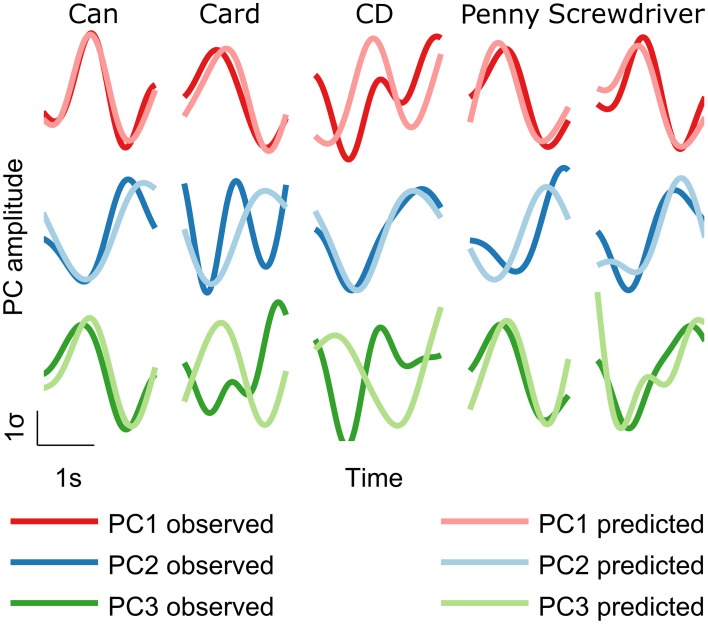
**Decoded trajectories**. Predicted trajectories (light traces) showed similarity with measured trajectories (heavy traces). Examples of decoded PC trajectories for each object are shown for subject S5.

Decoding accuracies were quantified as the median correlation coefficient between the predicted and measured kinematics across cross validation folds. For each subject, we calculated decoding accuracies across the best n channels, with n ranging from 1 to 58, to evaluate the dependence of decoding accuracy on the number of EEG channels used. The common pattern across all decoded kinematics (PCs and joint angle velocities) was a rapid initial increase followed by saturation/slow decrease (Figure 3). To determine the peak in a robust manner, we fit a double exponential to the curves. For PC1, PC2, and PC3, peak decoding accuracies (mean ± s.e.m) were r = 0.59 ± 0.04, 0.47 ± 0.06, and 0.32 ± 0.05, with the peaks occurring when 22, 29, and 27 EEG channels were used to decode, respectively (optimal number of channels for each subject are shown in Supplementary Materials Table S1). Examples of decoded PC trajectories are shown in Figure 4. For individual joint angle velocities, we found that peak decoding accuracies were highest for the index, middle and ring PIP joints (*r* = 0.65 ± 0.03, 0.63 ± 0.02, and 0.58 ± 0.02) and lowest for thumb CMC, index MCP and middle MCP joints (*r* = 0.38 ± 0.04, 0.39 ± 0.03, and 0.37 ± 0.02). The mean decoding accuracy across all joints was *r* = 0.49 ± 0.02. The decoding accuracies for joint angles and kinematic PCs are comparable, indicating that both kinematic spaces may be equally encoded in EEG-based sensor space. These decoding accuracies are comparable to results from ECoG studies (*r* = 0.52 for first synergy; Acharya et al., 2010) and intracortical studies in monkeys (*r* = 0.62 for grasp aperture and *r* = 0.46 for aperture velocity; Zhuang et al., 2010).

Decoding accuracies were highly significant (*p* < 0.001; Bonferroni corrected for multiple comparisons across all subjects, kinematic variables, number of EEG channels and cross validation folds). We also calculated empirical chance levels using two methods: (1) by scrambling the phase of the EEG (Theiler et al., 1992) and (2) by scrambling the EEG trial indices (Antelis et al., 2013). “Phase-scrambled” EEG signals were obtained by randomizing the phase in the Fourier domain, while keeping the magnitude unchanged, followed by a transformation back to the time domain. The assumption behind time-domain decoding is that EEG signals are phase-locked to the kinematics, and a randomization of the phase would theoretically result in zero decoding accuracy. In the case of “fold-scrambled” EEG, the pairing between EEG and kinematics across trials was randomized, so that EEG corresponding to the kinematics from trial *n* was assigned to trial *m*. The expected decoding accuracy in this case is also close to zero as EEG for one type of grasped object would correspond to kinematics of a different object. The decoding procedure as detailed in the Materials and Methods Section was applied to the “phase-scrambled” and “fold-scrambled” EEG signals with 5 random repetitions each. We found close to zero decoding accuracy in both cases (Figure 3), showing that the decoding accuracy obtained without scrambling is significantly higher than chance levels.

### Artifacts did not aid decoding

EEG is known to be affected by ocular and muscular artifacts (Goncharova et al., 2003), which may contribute to decoding if they are task-correlated. In our experiment, muscular artifacts are unlikely to affect decoding results because we low-pass filtered EEG signal at 1 Hz, and the dominant frequency content of muscular artifacts is above 8 Hz (Goncharova et al., 2003). Additionally, we excluded from analysis six peripheral EEG channels most likely to be affected by muscular artifacts (M1, M2, TP9, TP10, PO9, and PO10). We prevented ocular artifacts from affecting our results in two stages: (1) by using Independent Component Analysis (ICA) to identify and remove ocular artifacts (Delorme and Makeig, 2004), as detailed in the Materials and Methods Section, and (2) by experiment design: All objects to be grasped were presented to subjects in the same spatial location, likely resulting in identical eye movements for all objects, making it unlikely that such a common pattern across objects could distinguish between them. To show the efficacy of these steps, we ran the decoding procedure without the four frontal channels affected most by ocular artifacts (FP1, FP2, AF7, and AF8). The decoding accuracy was unchanged (Figure 3), demonstrating that ocular artifacts did not affect our decoding results.

### Neural representation of grasping kinematics in sensor space

**Figure 5:**
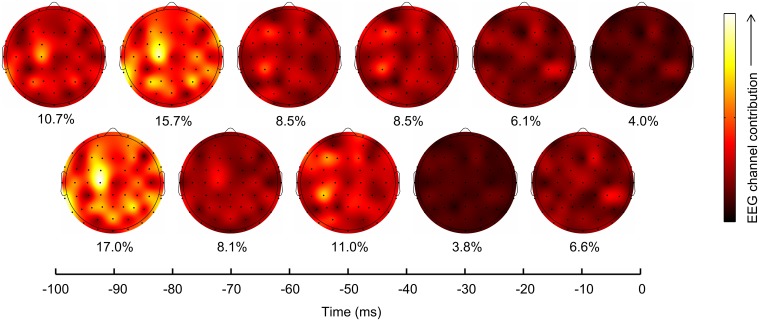
**Scalp locations and lags contribution to PC1 prediction**. EEG channel contributions to prediction of PC1 trajectory (mean across subjects) were plotted on scalp maps, with each map corresponding to a lag as indicated on the horizontal axis. The overall percentage contribution of each lag is shown below each scalp map. Lags −90 and −80 ms contributed maximally, particularly with EEG channels C1, FC1, P1, and P3. Channel CP5 was also a major contributor at other lags. Interestingly, all these channels lie above the known contralateral motor areas of the cortex.

To assess which scalp regions contributed the most to kinematics prediction, we plotted the contribution of EEG channels (see Section Materials and Methods) at each time lag on a scalp map, for PC1 (Figure 5), as PC1 accounted for 50 ± 1% of the joint velocity variance and was also the best decoded synergy. Lags −90 and −80 ms contributed maximally (17.0 and 15.7%), particularly at scalp locations C1, FC1, P1, and P3. Interestingly, C1 and FC1 are located above the primary motor cortex and supplementary motor areas, which may explain their high contributions. P1 and P3 lie above the associative cortices which process the visuo-motor transformations necessary for a reach-and grasp movement. In agreement with established neuroscience findings, the contralateral hemisphere made the highest contribution. However, motor areas from the ipsilateral hemisphere (C5, P5, and CP7) also made contributions. At around −60 to −40 ms, CP5 played the major role in decoding PC1 kinematics. Transient recruitment of specific EEG channels primarily between −90 and −40 ms argues against movement artifacts inadvertently aiding the decoding process, since movement artifacts are expected to correlate maximally with movement at zero lag.

### Grasp classification peaked 250 ms after movement onset

**Figure 6:**
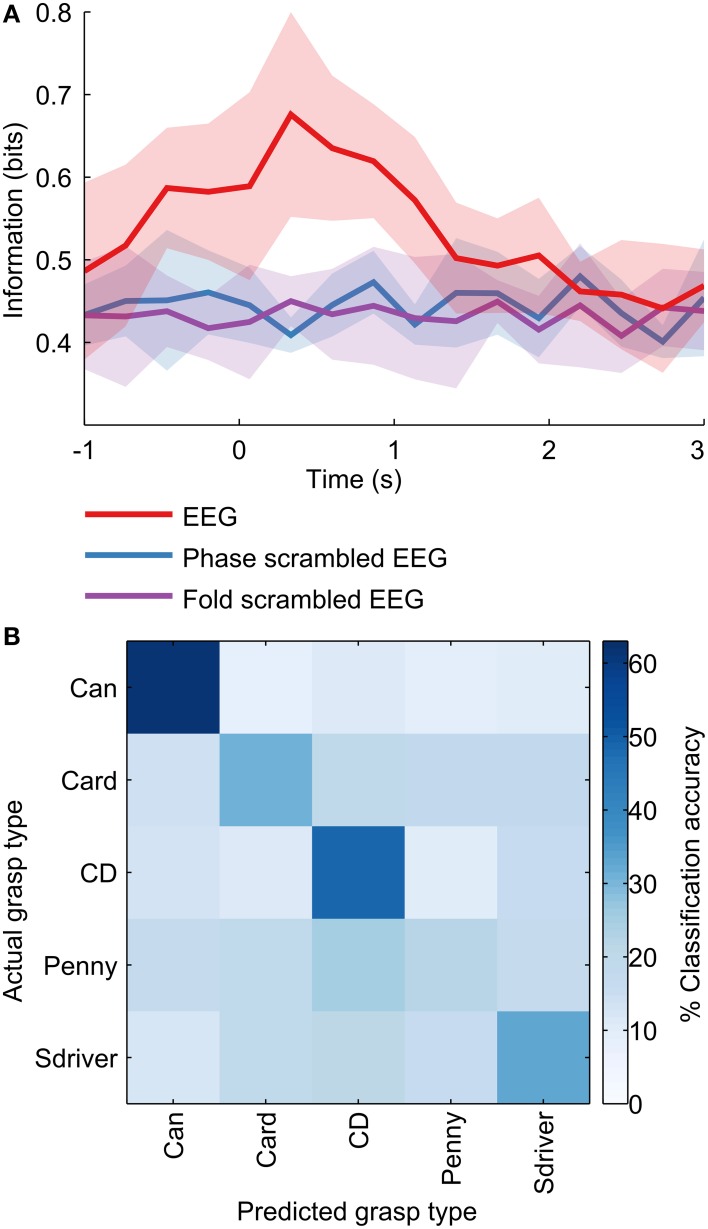
**Information content in EEG**. A discrete classifier predicted the object being grasped from 100 ms windows of preprocessed EEG at various points in time during the grasp. **(A)** The information content was calculated as the reduction in the entropy of the distribution over grasp types due to the classifier. Information in EEG peaked at around 250 ms after movement onset. **(B)** The classifier confusion matrix at 250 ms after movement onset is diagonal, indicating high classification accuracies. Precision grasps were classified with a lower accuracy (27%; card, penny) than whole-hand grasps (48%; can, CD, screwdriver). The overall decoding accuracy was 40% across the 5 objects, with chance level at 20%.

The decoding accuracies shown represent the prediction of the finger joint velocities/PCs, and can be implemented directly to control the grasping motion of a prosthetic hand. A hybrid approach could conceivably be used as well, in which the grasp type is predicted as a discrete class from EEG, based on which a pre-determined grasp trajectory can be implemented. A metric that measures the performance of such a classification-based motor prosthesis is the information (in bits) conveyed about the grasp type. We constructed MKL classifiers (see Materials and Methods) to classify 100 ms windows of EEG into discrete grasp types. When applied over the grasp duration, from −1 to 3 s with respect to movement onset, the information content in EEG peaked at 250 ms (Figure 6). This is in agreement with a previous study (Pistohl et al., 2012) which showed similar results with ECoG (electrocorticographic) data over a two-class grasp (precision vs. power) problem. For our five-class problem, the maximum information was 0.68 bits, occurring 250 ms after movement onset. The mean confusion matrix across subjects at 250 ms is shown in Figure 6. The classifier confusion matrix at 250 ms after movement onset is diagonal, indicating high classification accuracies. Precision grasps were decoded at a lower classification accuracy (27%; card, penny) than whole-hand grasps (48%; can, CD, screwdriver). Surprisingly, a penny (precision grasp) was often misclassified as a CD (whole hand circular grasp), possibly due to the similarity in their kinematic trajectory shapes (Figure 2A), despite the differences in amplitude. The overall decoding accuracy was 40% across the 5 objects, with chance level at 20%.

## Discussion

### Delta-band time domain EEG encodes grasping kinematics

Recent studies on monkeys and humans attempted to decode various aspects of grasping such as joint angles or grasp types from brain activity recorded through microelectrode arrays implanted in the brain or electrocorticographic (ECoG) grids placed over the cortex (Artemiadis et al., 2007; Hamed et al., 2007; Aggarwal et al., 2008; Kubánek et al., 2009; Acharya et al., 2010; Saleh et al., 2010; Vargas-Irwin et al., 2010; Zhuang et al., 2010; Agashe and Contreras-Vidal, 2011; Townsend et al., 2011; Pistohl et al., 2012). Activity of multiple neurons in the motor cortex has been shown to classify finger and wrist movements as well as grasp patterns (Artemiadis et al., 2007; Hamed et al., 2007; Aggarwal et al., 2008). Interestingly, individuated finger movements and movements during slow grasping motion can be predicted from the fluctuations of low-pass filtered (the so-called “local motor potential” or LMP) ECoG activity in humans (Kubánek et al., 2009; Acharya et al., 2010). Accurate classification of precision vs. whole-hand grip has also been shown with ECoG LMP (Pistohl et al., 2012). A recent study showed that motor networks controlling the upper limb exhibit an intrinsic periodicity at submovement frequencies in the delta band (0.1–4 Hz) that is reflected in the speed profile of movements (Hall et al., 2014). The present results, obtained using low-frequency time domain features, suggest that such an encoding mechanism, based on amplitude modulation, is observed in non-invasively recorded macro-scale level brain activity as well (Figure 3).

### Relevance to clinical populations and brain-machine interfaces

The spatial locations of highly contributing electrodes over multiple lags over the scalp suggest early recruitment of the contralateral frontal-central scalp areas and parietal electrodes, followed by involvement of the central electrodes over primary sensorimotor cortical areas (Figure 5). This pattern of spatiotemporal information processing is in agreement with previous studies (Castiello, 2005). Changes in these spatial patterns of neural activity, at the level of scalp electrodes, may provide a window to investigate the plasticity of the brain during learning to use a brain-machine interface (BMI). These maps of predictive electrodes may also be informative when compared to those from clinical populations. The high values and significance levels of the decoding accuracies (Figure 3) argue against the need for more localized means of extracting neural activity for decoding, and suggest that information about dexterous grasping movements are represented in fast-changing global networks at the EEG scale. Importantly, these findings merit further investigation to assess the feasibility of EEG-based decoding for closed-loop BMI systems to control dexterous neuroprosthetics.

The high decoding accuracies (compared to similar previous studies, see Table 1) obtained in this study suggest that this methodology is a promising candidate for application in real-time closed loop BMI systems for inferring desired grasping movements. We obtained similar levels of decoding for individual joint angles and synergies based on PCs, which suggests that a PC-based control scheme requiring lesser degrees of freedom is advantageous over individual joint angle control for closed-loop control of a hand neuroprosthesis. We are cognizant that effective BMI systems require decoding of movement intent in the absence of real movement. In this regard, recent studies demonstrated reach and grasp by tetraplegics using a neurally (intracranial electrodes) controlled robotic arm, albeit not as fast or accurate as those of an able-bodied person (Collinger et al., 2012; Hochberg et al., 2012; Wodlinger et al., 2015). Although the present study deciphers the cortical EEG signatures of actual movement, it is likely that some neural characteristics or features may be shared between imagined and real movements (Yuan et al., 2010; Bradberry et al., 2011; Hochberg et al., 2012; Agashe and Contreras-Vidal, 2013). Results from a few studies suggest that with training, patients could regain control of neural populations that would otherwise participate in natural movements for the purpose of a BMI (Hochberg et al., 2006, 2012; Collinger et al., 2012; Wodlinger et al., 2015). Our methodology could also help to elucidate the changes in the neural representation for movement during skill learning or during intervention to rehabilitate fine motor control after brain injury. Importantly, our results challenge the perceived limitations of scalp EEG as a source signal for BMI systems or their use to investigate cortical plasticity during imagined or performed motor acts.

### Demonstration of real-time closed-loop neuroprosthetic control of grasping by an amputee

**Figure 7:**
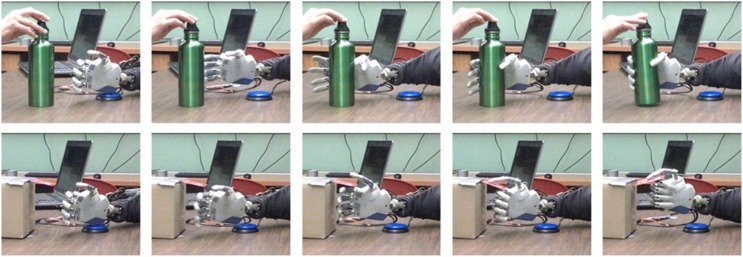
**Grasp pre-shaping with closed loop real-time hand neuroprosthetic control based on EEG**. Examples of successful grasps during the closed-loop control are shown for the cylindrical (**top row**) and the lateral (**bottom row**) grasps. Videos of the shown task are available in the Supplementary Section.

To investigate the feasibility of these methods for real-time closed-loop control of a hand neuroprosthesis, we implemented our methods to control neuroprosthetic grasping from EEG of an amputee participant (56 year old male). The participant was fitted with an anthropomorphic neuroprosthetic hand (IH2 Azurra, Prensilia s.r.l., Italy). During the training phase, the participant was instructed to reach out to grasp the presented object: either a bottle (cylindrical whole hand grasp) or a credit card (lateral precision grasp). Initiation of hand transport triggered a pre-determined grasping sequence in the robotic hand, suitable to the object being presented. The participant was instructed to imagine himself controlling the hand pre-shaping and grasping. In addition to the visual feedback, he was asked to imagine kinesthetic feedback as well. The grasp was held steady for 2 s, followed by an opening of the grasp and a return to the resting position (reverse of the grasping trajectory). During the grasp release trajectory, participants transported the hand back to its resting position (Figure 7). The participant performed 100 trials during the training phase. Following the training phase, EEG data and the pre-recorded hand kinematics were used to create a decoder, using methods similar to those described here in the Materials and Methods section. In the testing phase, the amputee participant was asked to reach and grasp the presented object by intending to perform such action within 5 s. The first and second Principal Components were controlled by the participant's EEG in real time. During the testing phase, the participant was able to achieve a grasping success rate of 80% over 100 trials. A video showing example grasps is available in the Supplementary Section.

In this study we decode with simple linear models as they have been shown to provide high decoding accuracies from a multitude of brain signals (spiking activity, LFP, ECoG, EEG, and MEG) (Bradberry et al., 2008, 2010; Acharya et al., 2010; Mollazadeh et al., 2011). Dexterous tasks like handwriting have been reconstructed from electromyography (EMG) signals from the hand with linear filters as well (Linderman et al., 2009). While evidence is mounting for a time-domain encoding mechanism in field potentials, including EEG, more research is needed to elucidate its relationship with frequency domain representations and spiking activity (Bansal et al., 2011). Further research is also required to characterize the consistency of the channel selections and neuroprosthetic control made across subjects and across sessions, and to investigate the role of higher frequencies for EEG decoding.

### Conflict of interest statement

The authors declare that the research was conducted in the absence of any commercial or financial relationships that could be construed as a potential conflict of interest.
