# Supplementary material for: Global cortical activity predicts shape of hand during grasping
Source: Front Neurosci. 2015 Apr 9;9:121. doi: 10.3389/fnins.2015.00121 (PMC4391035; doi:10.3389/fnins.2015.00121)
Supplement: Supplementary file 1 [file Table1.DOCX]

SUPPLEMENTARY MATERIAL

Table S1 **Optimal number of EEG channels for each subject**.

|  | Subject 1 | Subject 2 | Subject 3 | Subject 4 | Subject 5 |
| --- | --- | --- | --- | --- | --- |
| PC1 | 22 | 27 | 51 | 19 | 15 |
| PC2 | 54 | 35 | 28 | 19 | 34 |
| PC3 | 16 | 23 | 40 | 42 | 19 |

Antelis et al., 2013 recently discussed possible shortcomings of applying linear regression to low frequency time-domain EEG to predict kinematics. For our detailed response to this paper where we indicate the pitfalls of that method, please see our commentary posted online on plos one (Bradberry and Contreras-Vidal, 2013, “Comments concerning Antelis et al. 2013” available at <http://www.plosone.org/annotation/listThread.action?root=69163>).

Videos available in supplementary materials show examples of real-time closed-loop control of a neuroprosthetic to grasp either a bottle or a credit card, by an amputee.

**References**

Antelis, J. M., Montesano, L., Ramos-Murguialday, A., Birbaumer, N., and Minguez, J. (2013). On the usage of linear regression models to reconstruct limb kinematics from low frequency EEG signals. *PLoS One* 8, e61976. doi:10.1371/journal.pone.0061976.
